# Luteinizing hormone dynamics during progestin-primed ovarian stimulation do not predict follicular output in women with normo-ovarian reserve

**DOI:** 10.3389/fendo.2026.1825217

**Published:** 2026-05-19

**Authors:** Fazilet Kubra Boynukalin, Meral Gultomruk, Neslihan Sonmez, Peter Humaidan, Mustafa Bahceci, Gurkan Bozdag

**Affiliations:** 1 Infertility Department, Bahceci Fulya In Vitro Fertilization Center, Istanbul, Türkiye; 2 R&D Department, Bahceci Health Group, Istanbul, Türkiye; 3 Department of Clinical Medicine, Aarhus University, Aarhus, Denmark

**Keywords:** follicle-to-oocyte index, IVF (ICSI), LH, ovarian stimulation (OS), PPOS, suboptimal response

## Abstract

**Background:**

Progestin-primed ovarian stimulation (PPOS) has emerged as an effective alternative to conventional pituitary suppression strategies in assisted reproductive technology cycles. While suppression of luteinizing hormone (LH) during PPOS is well established, the clinical relevance of dynamic LH changes during stimulation remains unclear. This study aimed to investigate whether serial LH dynamics are associated with follicular output in women with non–low ovarian reserve undergoing PPOS.

**Methods:**

This retrospective cohort study included 616 ovarian stimulation cycles performed using a PPOS protocol with recombinant follicle-stimulating hormone (rFSH) between January 2019 and January 2025. Serum LH concentrations were measured at three predefined time points: baseline (cycle day 2–3), mid-stimulation (day 6–7), and trigger day. LH dynamics were assessed using calculated changes between time points (ΔLH1, ΔLH2, ΔLH3). Ovarian response was evaluated using the follicle-to-oocyte index (FOI), defined as the ratio of retrieved oocytes to antral follicle count (AFC). Patients were categorized as optimal responders (FOI ≥50%) or suboptimal responders (FOI <50%).

**Results:**

Among the 616 cycles analyzed, 549 patients (89.1%) demonstrated an optimal ovarian response, whereas 67 patients (10.9%) had a suboptimal response. Serum LH concentrations declined during ovarian stimulation in the overall cohort; however, LH levels were comparable between groups at all measured time points. Baseline LH levels were similar between optimal and suboptimal responders (7.21 ± 2.62 vs 7.13 ± 2.79 IU/L, p=0.86), as were mid-stimulation LH levels (6.43 ± 3.54 vs 6.43 ± 4.35 IU/L, p=0.99) and trigger-day LH levels (3.97 ± 2.75 vs 3.92 ± 2.41 IU/L, p=0.88). Dynamic LH changes during stimulation did not differ significantly between groups, including ΔLH1 (−0.77 ± 3.01 vs −0.70 ± 3.50 IU/L, p=0.843), ΔLH2 (−2.47 ± 2.78 vs −2.52 ± 3.13 IU/L, p=0.890), and ΔLH3 (−3.24 ± 2.78 vs −3.21 ± 2.40 IU/L, p=0.936). In multivariable logistic regression analysis, female age (OR 0.909, 95% CI 0.853–0.969, p=0.004) and AFC (OR 0.916, 95% CI 0.892–0.941, p<0.001) were independently associated with ovarian response, whereas LH-related parameters were not significant predictors of follicular output.

**Conclusion:**

In women with non–low ovarian reserve undergoing PPOS, serum LH levels decline during stimulation, but their absolute values and dynamic changes are not associated with follicular output.

## Introduction

The primary aim of ovarian stimulation (OS) in *in vitro* fertilization (IVF) and intracytoplasmic sperm injection (ICSI) cycles is to induce multifollicular development and retrieve mature oocytes while preventing spontaneous luteinization and ovulation. This strategy increases the number of available embryos, facilitates embryo selection, and improves overall reproductive outcomes. In the absence of pituitary suppression, premature luteinizing hormone (LH) surges occur in more than 20% of stimulation cycles and are associated with reduced metaphase II oocyte yield and lower pregnancy rates (1, 2). Traditionally, GnRH agonists or antagonists have been used to prevent premature LH surges during ovarian stimulation (3). More recently, progestin-primed ovarian stimulation (PPOS) has emerged as an alternative strategy for pituitary suppression during OS (4).

According to the 2025 ESHRE guideline on ovarian stimulation, routine hormonal monitoring—including luteinizing hormone (LH), estradiol (E2), and progesterone (P4)—in addition to ultrasound is not recommended because of its limited ability to predict clinical outcomes (5). Nevertheless, LH remains a key regulator of follicular steroidogenesis and follicular development during ovarian stimulation. Experimental and clinical evidence suggests that only a minimal level of LH activity is required to sustain normal follicular function. Even very low circulating LH concentrations may be sufficient to support estradiol production and continued follicular growth in the presence of exogenous gonadotropins. Indeed, LH levels as low as 0.5–1.2 IU/L have been reported to maintain adequate follicular steroidogenesis, reflecting the high sensitivity of ovarian LH receptors to circulating hormone levels (6).

Despite advances in ovarian stimulation strategies, a proportion of patients still exhibit a suboptimal ovarian response, characterized by a lower-than-expected oocyte yield despite apparently normal ovarian reserve parameters. This clinical heterogeneity has been further characterized by the POSEIDON classification system, which stratifies patients according to age, antral follicle count (AFC), anti-Müllerian hormone (AMH) levels, and previous ovarian stimulation outcomes (7). Among these patients, those with normal baseline ovarian reserve but unexpectedly low oocyte yield represent a particularly challenging subgroup in clinical practice. To better quantify this discrepancy, the follicle-to-oocyte index (FOI)—defined as the ratio between the number of retrieved oocytes and AFC—has been proposed as a marker of follicular efficiency (8). One possible explanation for a reduced FOI is an altered intrafollicular hormonal environment, particularly involving LH activity. LH stimulates androgen production in theca cells, which serves as a substrate for estradiol synthesis in granulosa cells and supports follicular growth and oocyte maturation. Excessive suppression of LH activity may therefore disrupt this hormonal cascade and impair follicular development. These considerations highlight the potential relevance of evaluating LH dynamics during OS, particularly in patients with discordant ovarian response patterns.

Although suppression of LH during PPOS has been well documented, most previous studies have focused on single or static LH measurements rather than evaluating dynamic hormonal changes throughout the stimulation period (9, 10). Consequently, the temporal pattern of LH fluctuations during PPOS and their potential relationship with ovarian response remain insufficiently characterized. The present study aimed to investigate whether serial LH dynamics differ between patients with suboptimal follicular response—defined by a low FOI—and those with optimal follicular output among women undergoing PPOS.

## Materials and methods

This retrospective cohort study included women who underwent ovarian stimulation using a PPOS protocol between January 2019 and January 2025. Eligible cycles involved stimulation with recombinant follicle-stimulating hormone (rFSH) preparations (follitropin alfa or follitropin beta) in combination with oral medroxyprogesterone acetate for pituitary suppression. Inclusion criteria were: (i) availability of serum luteinizing hormone (LH) measurements at three predefined time points (baseline: cycle day 2–3, mid-stimulation: day 6–7, and trigger day); (ii) female age ≤45 years; (iii) an initial rFSH dose of 225–300 IU/day; and (iv) evidence of non–low ovarian reserve defined by at least two consistent antral follicle count (AFC) measurements ≥5.

Cycles were excluded if they involved: (i) stimulation with human menopausal gonadotropin (HMG) alone or in combination with rFSH or recombinant LH; (ii) an initial rFSH dose <225 IU/day; (iii) missing LH measurements at any of the three predefined time points; (iv) the use of adjuvant agents during stimulation (e.g., growth hormone, clomiphene citrate, or aromatase inhibitors); (v) inconsistent or missing AFC data; or (vi) duplicate or overlapping cycles, in which case only one eligible cycle per patient was included in the analysis.

### OS protocol and oocyte retrieval

OS was performed using rFSH preparations (Gonal-F, Merck Serono, Germany or Puregon, Organon, Netherlands) at an initial dose of 225–300 IU/day. The initial rFSH dose (225–300 IU/day) was determined based on routine clinical practice and individualized according to patient characteristics, including age, body mass index, AFC, and previous ovarian response, at the discretion of the treating physician. Oral medroxyprogesterone acetate (MPA; Tarlusal, Deva, Turkey) was administered at a total daily dose of 10 mg from the first day of stimulation until the day of final oocyte maturation in order to prevent a premature LH surge.

Follicular development was monitored by serial transvaginal ultrasound examinations. Final oocyte maturation was triggered when at least two follicles reached a mean diameter of ≥18 mm. Triggering was performed using either 250 μg recombinant human chorionic gonadotropin (hCG) (Ovitrelle, Merck Serono, Germany) or 0.2 mg triptorelin acetate (Gonapeptyl, Ferring, Germany), according to the attending physician’s preference.

Transvaginal ultrasound-guided oocyte retrieval was performed 34–36 hours after trigger. All follicles with a mean diameter of ≥10 mm were aspirated using a single-lumen needle according to standard clinical practice.

### Assessments and hormone assays

Follicular development was monitored using serial transvaginal ultrasound examinations during ovarian stimulation. Serum concentrations of LH, E2, and P4 were measured at three predefined time points: baseline (cycle day 2–3), mid-stimulation (day 6–7 of stimulation), and on the day of final oocyte maturation trigger.

Hormone measurements were performed using an electrochemiluminescence immunoassay (ECLIA) with the Elecsys^®^ LH kit (Roche Diagnostics GmbH, Mannheim, Germany) on the cobas e 801 analyzer. To minimize the potential impact of diurnal hormonal variation, all blood samples were collected between 10:00 and 12:00 a.m.

### Outcome measures

The primary outcome of the study was to evaluate whether serum LH dynamics during ovarian stimulation differed between patients with suboptimal and optimal follicular responses during a PPOS protocol.

LH dynamics were assessed using serial serum LH measurements obtained at three predefined time points: baseline (cycle day 2–3), mid-stimulation (day 6–7), and the trigger day. Changes in LH concentrations were calculated as follows: ΔLH1 (mid-stimulation minus baseline), ΔLH2 (trigger day minus mid-stimulation), and ΔLH3 (trigger day minus baseline).

Ovarian response was evaluated using the FOI, defined as the ratio of the number of retrieved oocytes to the antral follicle count (AFC) multiplied by 100. A FOI value <50% was considered indicative of a suboptimal ovarian response, whereas FOI ≥50% represented an optimal response (8).

### Statistical analysis

All statistical analyses were performed using IBM SPSS Statistics for Windows, Version 27.0 (IBM Corp., Armonk, NY, USA). Continuous variables are presented as mean ± standard deviation (SD).

Patients were classified according to FOI into optimal responders (FOI ≥50%) and suboptimal responders (FOI <50%). Baseline demographic, clinical, hormonal, and laboratory parameters were compared between groups using the independent samples t-test. Differences in ΔLH parameters between the two groups were evaluated using the independent samples t-test.

To identify independent predictors of optimal ovarian response, a multivariable logistic regression analysis was performed with FOI category (optimal vs. suboptimal response) as the dependent variable. Clinically relevant continuous covariates, including female age, body mass index (BMI), AFC, total gonadotropin dose, stimulation duration, and LH-related parameters, were entered simultaneously into the model. Results are reported as regression coefficients (B), odds ratios (OR), and 95% confidence intervals (CI).

Visualization of LH trajectories across stimulation stages was performed using RStudio (Version 2025.05.1, Build 513; Posit Software, PBC) within the R statistical computing environment and was used solely for figure generation.

All statistical tests were two-tailed, and a p-value <0.05 was considered statistically significant.

## Results

A total of 616 OS cycles performed using a PPOS protocol met the inclusion criteria and were included in the final analysis. According to the FOI, 549 patients (89.1%) demonstrated an optimal ovarian response (FOI ≥50%), whereas 67 patients (10.9%) exhibited a suboptimal response (FOI <50%).

Baseline demographic, hormonal, and stimulation characteristics of the study population are presented in **Table 1**. Female age and baseline serum LH levels were comparable between patients with FOI ≥50% and those with FOI <50% (28.8 ± 4.3 vs. 29.9 ± 4.7 years, p = 0.12; 7.2 ± 2.6 vs. 7.1 ± 2.8 IU/L, p = 0.86). Body mass index (BMI) was slightly higher in the FOI <50% group (27.4 ± 5.1 vs. 25.6 ± 4.7 kg/m², p = 0.018). Interestingly, despite having a significantly higher antral follicle count (AFC) (28.9 ± 11.4 vs. 19.1 ± 8.5, p < 0.001) and higher anti-mullerian hormone (AMH) (4.9 ± 1.9 vs. 3.4 ± 1.8, p=0.04), patients with FOI <50% yielded fewer total oocytes (11.2 ± 4.2 vs. 20.2 ± 8.5, p < 0.001) and metaphase II (MII) oocytes (9.4 ± 3.9 vs. 16.2 ± 7.4, p < 0.001). Total gonadotropin dose and stimulation duration were comparable between the two groups. Final estradiol levels (3042.3 ± 2786.3 vs. 4092.4 ± 2868.1 pg/mL, p = 0.003) and progesterone levels (0.95 ± 0.75 vs. 1.22 ± 1.05 ng/mL, p = 0.041) were significantly lower in patients with FOI <50%.

**Table 1:** Baseline characteristics of the study population according to ovarian response.

Variable	Total (N = 616) mean ± SD	FOI ≥50% (n = 549) mean ± SD	FOI <50% (n = 67) mean ± SD	p
Female age (years)	28.94 ± 4.37	28.83 ± 4.31	29.85 ± 4.73	0.12
BMI (kg/m²)	25.77 ± 4.76	25.57 ± 4.68	27.40 ± 5.13	**0.018**
Total gonadotropin dose (IU)	2488.11 ± 480.31	2492.24 ± 480.96	2454.29 ± 477.12	0.59
AFC	20.19 ± 9.38	19.13 ± 8.54	28.88 ± 11.35	**<0.001**
AMH (ng/ml)	3.8 ± 2	3.4 ± 2	4.9 ± 1.9	**0.04**
Initial E2 (pg/mL)	50.44 ± 75.39	51.10 ± 79.54	45.11 ± 19.97	0.53
Initial P4 (ng/mL)	0.27 ± 0.19	0.28 ± 0.19	0.27 ± 0.19	0.71
Initial LH (IU/L)	7.20 ± 2.64	7.21 ± 2.62	7.13 ± 2.79	0.86
Mid-cycle E2 (pg/mL)	1676.48 ± 1314.59	1706.54 ± 1317.59	1430.13 ± 1272.66	0.15
Mid-cycle P4 (ng/mL)	0.67 ± 0.70	0.68 ± 0.73	0.55 ± 0.46	0.18
Mid-cycle LH (IU/L)	6.43 ± 3.63	6.43 ± 3.54	6.43 ± 4.35	0.99
Final E2 (pg/mL)	3978.15 ± 2875.75	4092.37 ± 2868.08	3042.27 ± 2786.25	**0.003**
Final P4 (ng/mL)	1.19 ± 1.02	1.22 ± 1.05	0.95 ± 0.75	**0.041**
Final LH (IU/L)	3.96 ± 2.71	3.97 ± 2.75	3.92 ± 2.41	0.88
Number of COC	19.25 ± 8.57	20.24 ± 8.5	11.18 ± 4.19	**<0.001**
No of MII	15.45 ± 7.4	16.19 ± 7.39	9.39 ± 3.89	**<0.001**

p-values were calculated using the independent samples t-test. FOI, follicle-to-oocyte index; BMI, body mass index; AFC, antral follicle count; E2, estradiol; P4, progesterone; LH, luteinizing hormone; COC, cumulus–oocyte complex; MII, metaphase II.

Values presented in bold indicate statistical significance.

Serum LH concentrations declined progressively throughout the stimulation period across the entire cohort. However, LH levels were comparable between the two groups at each measured time point, including mid-stimulation and trigger day. Similarly, the dynamic changes in LH levels did not differ significantly between patients with suboptimal and optimal ovarian responses. From baseline to mid-stimulation, ΔLH1 was −0.77 ± 3.01 IU/L in the FOI ≥50% group and −0.70 ± 3.50 IU/L in the FOI <50% group (p = 0.843). From mid-stimulation to trigger day, ΔLH2 was −2.47 ± 2.78 IU/L and −2.52 ± 3.13 IU/L, respectively (p = 0.890). The overall change in LH levels from baseline to trigger day (ΔLH3) was also similar between groups (−3.24 ± 2.78 vs. −3.21 ± 2.40 IU/L, p = 0.936). These findings indicate that LH dynamics during ovarian stimulation were comparable regardless of follicular response status (**Table 2**).

**Table 2:** Comparison of delta LH changes between suboptimal and optimal responders.

Variable	Group	N	Mean ± SD	p-value
ΔLH1 (mid − initial)	Suboptimal	67	−0.70 ± 3.50	
	Optimal	549	−0.77 ± 3.01	0.843
ΔLH2 (final − mid)	Suboptimal	67	−2.52 ± 3.13	
	Optimal	549	−2.47 ± 2.78	0.890
ΔLH3 (final − initial)	Suboptimal	67	−3.21 ± 2.40	
	Optimal	549	−3.24 ± 2.78	0.936

ΔLH was calculated as the difference between measurements: ΔLH1 = Mid − Initial, ΔLH2 = Final − Mid, ΔLH3 = Final − Initial. Values are presented as mean ± SD.

**Figure 1:**
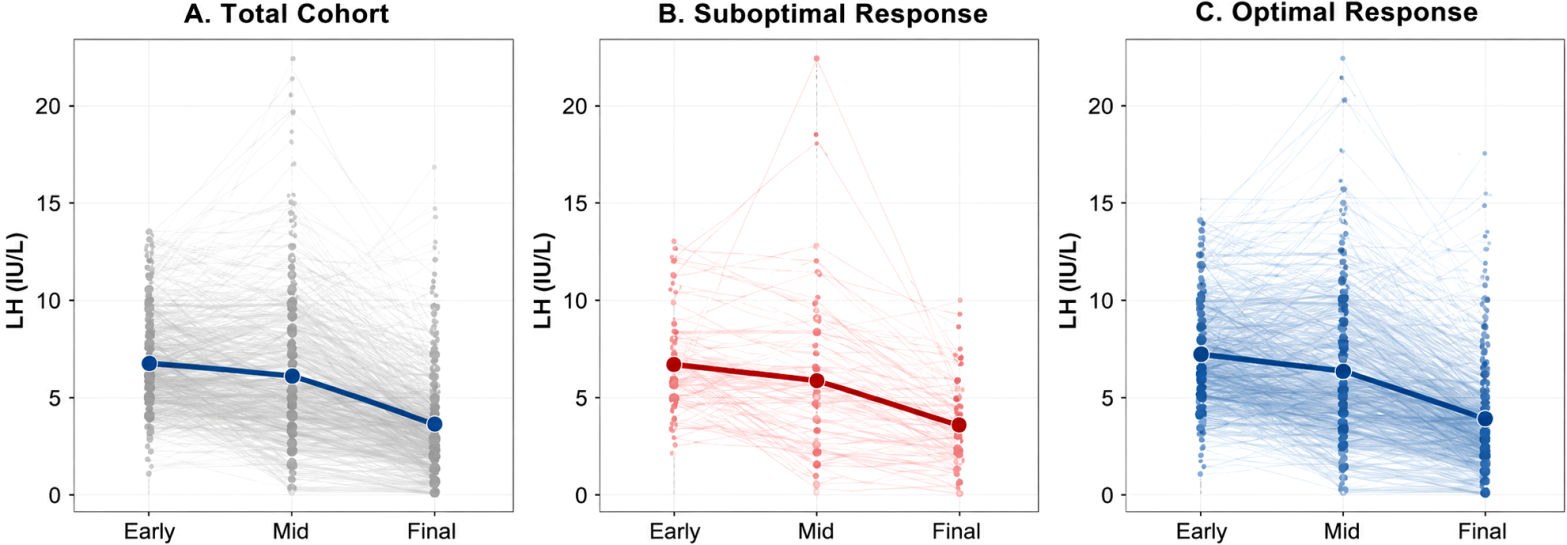
LH dynamics across monitoring stages.

The trajectory of serum LH levels across the stimulation period is illustrated in **Figure 1** A gradual decline in LH concentrations was observed from baseline to the trigger day in the overall cohort (**Figure 1A**). Similar patterns were observed in both the FOI <50% group (**Figure 1B**) and the FOI ≥50% group (**Figure 1C**), further demonstrating the absence of meaningful differences in LH dynamics between patients with suboptimal and optimal ovarian responses.

Multivariable logistic regression analysis was performed to identify independent predictors of optimal ovarian response (FOI ≥50%). Female age (OR: 0.909, 95% CI: 0.853–0.969, p = 0.004) and AFC (OR: 0.916, 95% CI: 0.892–0.941, p < 0.001) were independently associated with achieving an optimal response (**Table 3**). Increasing female age and higher AFC values were associated with a lower probability of achieving FOI ≥50%. In contrast, BMI, total gonadotropin dose, stimulation duration, and LH-related parameters were not significant predictors in the multivariable model.

**Table 3:** Multivariable logistic regression predicting FOI.

Variable	B	SE	Wald	p-value	OR	95% CI
Female age (years)	−0.095	0.033	8.477	**0.004**	0.909	0.853–0.969
BMI (kg/m²)	−0.053	0.035	2.242	**0.134**	0.949	0.886–1.016
AFC	-0.088	0.014	41.615	<0.001	0.916	0.892-0.941
Total gonadotropin dose (IU)	0.000	0.000	0.601	0.438	1.000	0.999-1.001
Duration of stimulation (days)	-0.068	0.163	0.174	0.677	0.934	0.679-1.286
Initial LH (IU)	0.076	0.062	1.474	0.225	1.079	0.955–1.219
Final LH (IU)	-0.008	0.061	0.017	0.897	0.992	0.880–1.118
Constant	7.758	2.046	14.382	<0.001	—	—

Multivariable logistic regression was performed on the full cohort (N = 616). The dependent variable was suboptimal response (FOI <50%) vs optimal response (FOI ≥50%). Results are presented as regression coefficient (B), standard error (SE), Wald statistic, odds ratio (OR), and 95% confidence interval (CI). FOI, follicle-to-oocyte index; BMI, body mass index; AFC, antral follicle count; LH, luteinizing hormone; CI, confidence interval.

Values presented in bold indicate statistical significance.

In the subgroup analysis, patients with PCOS were younger and had higher BMI, AFC, and LH levels (baseline, mid-cycle, and final) compared to non-PCOS patients (all p < 0.05). The number of retrieved oocytes was also significantly higher in the PCOS group (p < 0.001). However, ΔLH parameters were comparable between groups (all p > 0.05). Notably, the proportion of patients with suboptimal follicular output (FOI <50%) was significantly higher in the PCOS group (14.5% vs. 9.5%, p = 0.041) (**Table 4**).

**Table 4:** Comparison of LH dynamics and follicular output (FOI) according to PCOS status.

Variable	PCOS (n = 165) Mean ± SD	Non-PCOS (n = 451) Mean ± SD	p
Female age (years)	28.28 ± 3.9	29.18 ± 4.5	0.024
BMI (kg/m²)	27.94 ± 5.28	24.97 ± 4.28	<0.001
Total gonadotropin dose (IU)	2394 ± 470	24576± 477	0.718
AFC	26.3 ± 6.13	18.31 ± 7.89	<0.001
Initial LH (IU/L)	8.5 ± 2.97	6.9 ± 2.49	0.042
Mid-cycle LH (IU/L)	7.4 ± 4	6.2 ± 3.5	0.011
Final LH (IU/L)	3.8 ± 2.9	3.2 ± 2.6	0.022
Number of COC	23.6 ± 9.3	17.7 ± 7.7	<0.001
ΔLH1	-1.2 ± 3.1	-0.89 ± 3.1	0.11
ΔLH2	-2.8 ± 2.9	-2.3 ± 2.7	0.13
ΔLH3	-4.1± 2.7	-3.2 ± 2.7	0.875
FOI			0.041
FOI <50%	24 (14.5%)	43 (9.5%)	
FOI ≥50%	131 (85.5%)	408 (90.5%)	

Values are presented as mean ± standard deviation (SD) or number (percentage), as appropriate. Comparisons between groups were performed using the independent samples t-test for continuous variables and the chi-square test for categorical variables. FOI (follicle-to-oocyte index) was defined as the ratio of retrieved oocytes to antral follicle count (AFC) multiplied by 100. ΔLH values represent changes between time points (ΔLH1: mid-stimulation minus baseline; ΔLH2: trigger day minus mid-stimulation; ΔLH3: trigger day minus baseline). A p-value <0.05 was considered statistically significant.

## Discussion

The present study investigated whether serum LH dynamics during PPOS are associated with follicular output in women with non–low ovarian reserve undergoing IVF/ICSI. Our findings demonstrated that neither absolute LH levels nor their dynamic changes throughout stimulation differed between patients with suboptimal and optimal follicular responses. These results suggest that serial LH monitoring during PPOS does not provide additional predictive value for identifying patients at risk of suboptimal follicular output in normo-responder populations.

In the multivariable analysis, female age and AFC emerged as independent predictors of follicular output. Increasing female age was associated with a reduced likelihood of achieving an optimal FOI, which is consistent with the well-established decline in ovarian responsiveness with advancing age. With increasing age, granulosa cell FSH receptor density and sensitivity are known to decrease, which results in reduced ligand–receptor binding efficiency and impaired receptor-mediated clearance of circulating FSH (11). This decline in receptor function may underlie the observed reduction in follicular responsiveness with advancing age. Moreover, aging is associated with a reduction in functional LH receptors in theca cells, as well as decreased biological activity of endogenous LH (12, 13).

High AFC may not necessarily reflect improved follicular efficiency but rather an increased pool of heterogeneous follicles, many of which may exhibit reduced sensitivity to gonadotropins. Elevated AMH levels, commonly observed in such patients, may further inhibit follicular recruitment and contribute to reduced follicle-to-oocyte conversion efficiency. In the context of PPOS, where LH levels are pharmacologically modulated, our findings suggest that intrinsic ovarian factors rather than LH dynamics may predominantly determine follicular output. Interestingly, AMH levels were significantly higher in patients with suboptimal follicular output. This finding may suggest that, despite a quantitatively higher ovarian reserve, increased AMH levels could be associated with reduced follicular recruitment efficiency, potentially contributing to a lower FOI. This observation is consistent with previous studies reporting an inverse relationship between AMH levels and follicular output efficiency (14). Furthermore, evidence from large cohorts suggests that women with high AFC may still demonstrate poor oocyte retrieval efficiency, possibly due to disrupted FSH surge dynamics, as observed in PCOS patients following GnRHa triggering (15). These findings raise the possibility that a high follicle count may reflect quantitative reserve but not necessarily efficient recruitment and may even impair oocyte yield in certain hormonal contexts.

In both polycystic ovary syndrome and poor ovarian reserve populations, several studies have demonstrated that PPOS protocols using different progestins—such as MPA and dydrogesterone—provide consistent and effective suppression of endogenous LH levels throughout stimulation (16, 17). These findings confirm the reliability of progestin-based pituitary suppression even in hormonally heterogeneous groups. However, while oocyte yield, maturation, and fertilization rates have been widely reported and found to be comparable across various PPOS strategies, the evaluation of suboptimal responses—particularly through objective markers such as FOI—has been largely overlooked in these populations. As a result, despite adequate LH suppression, the potential relationship between LH dynamics and follicular inefficiency remains underexplored in both high- and low-responder phenotypes.

In a previous study, three different protocols for pituitary suppression were compared, and the PPOS group of patients—particularly those receiving MPA—had significantly lower LH levels on the trigger day compared to the GnRH antagonist group of patients (18). However, in our study, as well as in other referenced reports (16, 17),, LH levels during the PPOS protocol were not as profoundly suppressed. This suggests that the extent of LH suppression observed in that study may not be consistently replicated across different cohorts, which highlights potential variability depending on study design, patient population, and clinical setting. Based on the study by Özcan et. al, LH supplementation might be considered in PPOS protocols (18). However, we found no significant difference in LH levels between patients with suboptimal and optimal responses. These findings suggest that LH dynamics may not be a major determinant of follicular output in normo-responder PPOS cycles. However, given that our study did not include patients receiving LH supplementation or those with profoundly suppressed LH levels, no definitive conclusions can be drawn regarding the potential benefit of LH supplementation in specific subgroups.

Several meta-analyses have reported improved clinical outcomes with LH activity supplementation during ovarian stimulation —either through HMG or rLH supplementation—compared to rFSH alone; however, beneficial effects have predominantly been demonstrated in the context of long GnRH agonist protocols (19, 20). The long GnRH agonist protocol is known to cause profound endogenous LH suppression. This profound LH suppression may explain the improved outcomes with LH supplementation in these protocols. Thus, the observed benefit of LH activity may be attributable to its role in compensating for the negative effects of excessive downregulation, highlighting the importance of maintaining a physiological LH threshold during stimulation. In this study, across the overall patient cohort, LH concentrations showed a progressive decline throughout the stimulation period (**Figure 1**). From baseline to mid-stimulation and further to the final day, a consistent downward trend was observed. Despite this gradual reduction, mean LH values remained within physiological ranges, and no critical suppression (<0.5 IU/L) was observed at the population level. In addition, at each time point—initial, mid-stimulation, and trigger day—no statistically significant differences were observed between the two groups. Similarly, the absolute changes in LH levels over time (from initial to mid-stimulation, initial to trigger, and mid to trigger) were comparable between groups, with no significant intergroup variation. Taken together, these graphical findings indicate that the dynamic pattern of LH secretion was similar regardless of follicular response, and thus LH levels or their changes during stimulation do not appear to predict the ovarian response of this cohort.

Given the nature of the PPOS protocol, a freeze-all strategy was uniformly applied in this study. As a result, any potential impact of LH levels on endometrial receptivity or implantation could not be assessed, since embryo transfer was deferred and endometrial function was not part of the outcome evaluation. Therefore, while LH dynamics may influence endometrial conditions in other contexts, this study specifically focused on the ovarian response, particularly aiming to identify clinical and hormonal predictors of suboptimal follicular output. Interpretation of findings should thus remain limited to OS parameters rather than broader reproductive outcomes such as implantation or live birth.

Given the observed influence of FSHR and LHCGR polymorphisms on OS outcomes, it becomes increasingly relevant to consider the genetic background of LH signaling pathways in clinical evaluation (21). Since LH plays a central role in follicular maturation and ovulation, baseline LH levels and their physiological impact may vary significantly depending on genetic variants, particularly those affecting LH receptor function or LH secretion dynamics. Therefore, incorporating genetic assessment of LH-related genes, such as *LHCGR* polymorphisms, could enhance personalized ovarian stimulation protocols. This approach may help clinicians identify patients at risk of suboptimal response and adjust gonadotropin dosing strategies accordingly, thereby optimizing treatment efficacy and minimizing unnecessary exposure to high rFSH doses.

Our findings showing comparable LH levels between suboptimal and optimal responders suggest that LH may not play a central role in determining follicular output in normo-responder PPOS cycles. This raises questions regarding the routine use of LH supplementation in this population. Evidence from *in vitro* maturation studies suggests that oocyte competence depends on preserved cumulus–oocyte communication via transzonal projections, which are physiologically withdrawn following LH-induced ovulatory signaling (22). Premature activation of this process may be detrimental, particularly in smaller follicles. However, as these data are derived from experimental invitro maturation settings, their relevance to conventional IVF remains unclear and should be interpreted with caution.

The study presents several methodological strengths. First, the inclusion of a well-defined and homogeneous patient population—women with normal ovarian reserve undergoing a standardized PPOS protocol with rFSH monotherapy—minimizes variability and strengthens internal validity. The serial assessment of LH levels at three clinically relevant time points (baseline, mid-stimulation, and trigger day) offers a dynamic perspective of hormonal fluctuations, enhancing the interpretability of LH-related findings. Furthermore, the use of FOI rather than oocyte count alone provides a more refined measure of ovarian response, allowing for better discrimination of suboptimal responders. The large sample size, along with stratified subgroup analysis, adds to the robustness of the conclusions. Finally, the study protocol closely mirrors real-world clinical practice, improving the applicability of the findings to everyday patient care.

Nonetheless, several limitations should be acknowledged. The retrospective design inherently limits the ability to infer causality and may introduce selection bias or unmeasured confounders. Although the event rate allowed multivariable analysis, no *a priori* sample size calculation was performed because of the retrospective study design. Due to the freeze-all policy mandated by the PPOS protocol, implantation and live birth outcomes could not be assessed, precluding conclusions about the impact of LH dynamics on endometrial receptivity or overall reproductive success. Moreover, reproductive and embryological outcomes are influenced by multiple additional factors beyond ovarian stimulation, including endometrial preparation protocols, embryo transfer strategies, paternal factors, and laboratory conditions. Therefore, inclusion of these outcomes could introduce substantial heterogeneity and potentially obscure the specific relationship between LH dynamics and ovarian response, which was the primary focus of this study. Additionally, LH levels were evaluated only in serum, which may not fully capture intrafollicular hormonal activity or LH receptor-mediated effects within the ovary. The findings are specific to the PPOS-rFSH setting and may not be generalizable to other stimulation protocols involving GnRH agonists, GnRH antagonists, or LH-containing regimens. Lastly, while the study identifies clinical patterns of suboptimal response, it does not explore mechanistic or molecular pathways underlying poor follicular recruitment, leaving important biological questions unanswered.

In conclusion, although LH levels declined progressively during stimulation in PPOS cycles, neither their absolute values nor their temporal changes were associated with suboptimal follicular response. These results suggest that LH dynamics alone may not be reliable predictors of ovarian efficiency in normo-responder populations. Future prospective studies incorporating endocrine and molecular profiling—such as intra-follicular hormone concentrations, granulosa cell gene expression, or LH receptor polymorphisms—may provide further insight into individual variability in OS response. Randomized trials assessing different LH supplementation strategies within PPOS protocols and studies integrating live birth data in freeze-all contexts are also warranted.

## Data Availability

The datasets presented in this study can be found in online repositories. The names of the repository/repositories and accession number(s) can be found below: https://doi.org/10.6084/m9.figshare.31673350.
